# Global Comparison of Erythrocyte EPA and DHA Concentrations in Pregnant Women

**DOI:** 10.1016/j.tjnut.2025.101299

**Published:** 2025-12-27

**Authors:** Tessa Deutsch, William S Harris, Kristina Harris Jackson, Andreas Hahn, Jan Philipp Schuchardt

**Affiliations:** 1 Institute of Food and One Health, Faculty of Natural Sciences, Foundation Leibniz University Hannover, Hannover, Germany; 2 The Fatty Acid Research Institute, Sioux Falls, SD, United States; 3 Department of Internal Medicine, Sanford School of Medicine, University of South Dakota, Sioux Falls, SD, United States

**Keywords:** omega-3 fatty acids (EPA/DHA), pregnancy nutrition, erythrocyte fatty acid, Omega-3 Index, maternal health

## Abstract

**Background:**

Adequate concentrations of long-chain omega-3 (LC n–3) PUFAs, specifically EPA and DHA, are critical for maternal health and fetal development during pregnancy. Despite their importance, global data on maternal blood concentrations of EPA+DHA remain sparse and inconsistent, partially due to differences in measurement methodologies.

**Objectives:**

This study assessed global maternal blood concentrations of EPA+DHA during pregnancy by synthesizing data from observational studies and RCTs from the last 20 y (2004–2025).

**Methods:**

Non–red blood cell (RBC) based EPA+DHA blood concentrations from published studies were standardized using conversion equations to estimate relative EPA+DHA percentages in RBCs [estimated Omega-3 Index (eO3I)]. Country mean eO3I levels were classified into 4 categories based on literature-defined thresholds.

**Results:**

An analysis of 66 studies involving 33,390 pregnant women from 28 countries revealed significant geographical disparities in eO3I levels. Only the Seychelles, Norway, and Ghana achieved desirable levels (>8%). Some Asian countries (Japan, Taiwan, and Singapore), Malawi, Tanzania, and Northern European nations (Belgium, Netherlands, Iceland, Denmark, and Sweden) exhibited sufficient/moderate levels (>6%–8%). Most countries, including the United States, Canada, Mexico, Brazil, Chile, Australia, the United Kingdom, Germany, Switzerland, Italy, Croatia, and Spain, demonstrated insufficient/low levels (>4%–6%). Meanwhile, China, India, and Iran showed very low/undesirable levels (≤4%).

**Conclusions:**

These findings highlight widespread insufficiency in maternal EPA+DHA status globally, with particularly severe deficiencies observed in Asia and parts of Europe. This study underscores the need for more research to ultimately define the optimal EPA+DHA concentrations during pregnancy using standardized blood biomarkers, along with pregnancy-specific reference ranges, to facilitate targeted nutritional strategies aimed at optimizing health outcomes for both mother and child. Future studies should focus on addressing data gaps, refining intake recommendations, and promoting accessible supplementation strategies.

## Introduction

Adequate supply of long-chain omega-3 polyunsaturated fatty acids (LC n–3 PUFAs), mainly EPA (C20:5) and DHA (C22:6), during pregnancy is crucial for not only fetal and infant development but also maternal health. Higher maternal blood concentrations of EPA and especially DHA are associated with better maternal and fetal/infant health outcomes such as lower risk of pre-eclampsia and preterm birth [[1], [2], [3], [4]], improved metabolic [5,6], cardiovascular [7], and mental health [8,9], as well as regulation of immune function [[10], [11], [12]] and brain and retina development [[13], [14], [15]].

Unlike α-linolenic acid (ALA, C18:3), EPA+DHA are not classified as essential fatty acids (FAs), as they can be synthesized from ALA. Several expert societies recommend that ALA should account for ∼0.5% to 1.2% of total daily energy intake [[16], [17], [18]]. However, the conversion of ALA to EPA, and particularly to DHA, is low and varies interindividually [19]. Although, estrogen increases the conversion of ALA into EPA or DHA by upregulating the conversion enzymes [20], the increase is not sufficient to meet fetal DHA needs. Thus, direct intake of preformed EPA and especially DHA from dietary sources (e.g. 1 portion of oily fish per week) or supplementation remains recommended for optimal pregnancy outcomes by numerous nutrition, gynecological, and pediatric societies [16,17,[21], [22], [23]]. The International Society for the Study of Fatty Acids and Lipids recommends 500 mg of EPA+DHA per day for the general adult population for cardiovascular health [24] and 200 mg of DHA additionally for pregnant women [25].

Assessing EPA+DHA supply often relies on dietary intake questionnaires [26,27], but this approach has inherent flaws due to biases (underreporting/overreporting), dependence on memory, inconsistencies or gaps in food databases, differences in cooking methods, and seasonal fluctuations in fish availability [28]. (A 7-item questionnaire assessing EPA+DHA intake was able to identify pregnant women who would benefit from high dose EPA+DHA supplementation to reduce preterm birth risk [29,30], but its utility may not translate to other countries or communities). Owing to the variability in dietary intake data, a more objective marker reflecting the biological status of EPA+DHA in the body may be a better tool to compare across studies, cultures, and diets and to use clinically [31].

Although there is good evidence for the importance of EPA+DHA in maternal and child health/development, there is a paucity of literature that has explicitly addressed the question of what the blood status of EPA+DHA is (and should be) in pregnant women country by country. This may be due to several factors. First, there are simply not enough studies done in pregnancy that have measured and reported blood FA values along with relevant clinical outcomes. Second, the available data on EPA+DHA blood status are reported in different blood fractions [e.g. red blood cells (RBCs), whole blood (WB), or various plasma lipid classes] and units (e.g. wt%, mol%, and μg/mL) [32], impeding a direct comparison of data across studies. Third, the studies that exist typically recruited women from a specific city or state and were not formal population-wide samplings. This forced us to extrapolate from potentially unique samples sets to entire countries.

The proportion of EPA and DHA relative to total FAs in RBCs—known as the Omega-3 Index (O3I)—is commonly used as an indicator of long-term EPA and DHA status in the body [33], since it reflects the mean EPA+DHA intake of several weeks [33] and has the lowest intraindividual variability [34]. To facilitate uniform comparison of EPA+DHA blood concentrations across different cohorts, conversion models have been used to estimate the O3I [estimated O3I (eO3I)] from various blood fractions and units [[35], [36], [37]].

Using this conversion rationale, the aim of this study is to provide a global overview of the eO3I in pregnant women using the data available from existing literature. We selected the eO3I as the focus of our analysis because this metric is well established for determining the EPA+DHA status [31,33,36,38,39]. We extracted data from observational studies (OSs) and randomized clinical trials (RCTs) published within the last 21 years and converted non–RBC-based EPA+DHA and DHA-only metrics to RBC-based metrics newly developed and recently published conversion equations [35,36]. Data from studies within the same countries were weighted and synthesized into a country-level estimate, then categorized using a color-coded system to visualize the regional variation in EPA+DHA status.

## Methods

### Literature search strategy

To identify relevant studies, a literature search was conducted between May 2024 and August 2025 using PubMed and the Clinical Study Database from the Global Organization for EPA and DHA Omega-3s (GOED). The search focused on identifying original research reports of OSs and RCTs (Supplementary Table 1). The study was conducted and reported in accordance with the PRISMA guidelines [40].

### Inclusion and exclusion criteria

Inclusion criteria were as follows:

• Publication language was English.
• Only articles with full text were included.
• EPA and DHA or only DHA levels (concentrations or relative amounts) in WB, RBCs, plasma total lipids (PTL), plasma phospholipid (PPL), or plasma phosphatidylcholine (PPC) measured by gas chromatography.
• Blood samples had to be drawn in 2004 or later. Studies were also included if blood sampling took place in 2004, even if the study started before that year. For studies that did not specify a sample collection year (typically RCTs), we first attempted to contact the authors. If this was unsuccessful, we assumed that any article published in 2008 or later used blood samples drawn in 2004 or thereafter.
• For RCTs, only baseline data were included.

Exclusion criteria were as follows:

• Studies that reported FA data only from other body fluids, tissues, or cells (e.g. cord blood, placenta, spinal fluid, muscle, platelets, and leukocytes).
• Publications where EPA and DHA concentrations were presented in unconvertable metrics (e.g. μg/g RBC).
• Studies whose subjects came from multiple countries and where the data could not be attributed to the corresponding country.

The study population was not limited by health status, dietary pattern (e.g. vegan, vegetarian, Mediterranean diet, etc.), or fish oil supplementation status. The objective was to describe the EPA+DHA status of pregnant women, not to determine the underlying causes of observed differences.

### Ethical considerations

As this work involved the synthesis of data from previously published data, no new human or animal subjects were involved, and ethical approval was not required. All primary studies included in this review stated that ethical approval had been obtained from the appropriate institutional review boards and that informed consent was received from participants, where applicable.

### Initial screening and data extraction

The search results were evaluated against the established inclusion and exclusion criteria. If the requisite information was not readily discernible from the initial examination of the publications, the complete texts were reviewed. The data on EPA and DHA concentrations, subject age, trimester, study type (RCT and OS), year of baseline data collection, and year of publication were extracted and transferred to a spreadsheet. Additionally, an examination was conducted to ascertain whether the EPA and DHA data from different publications originated from the same study that had been published on multiple occasions with varying research questions (duplicate study). In instances where this was the case, only the publication with the largest sample size was included in the analysis.

### Search results

**FIGURE 1:**
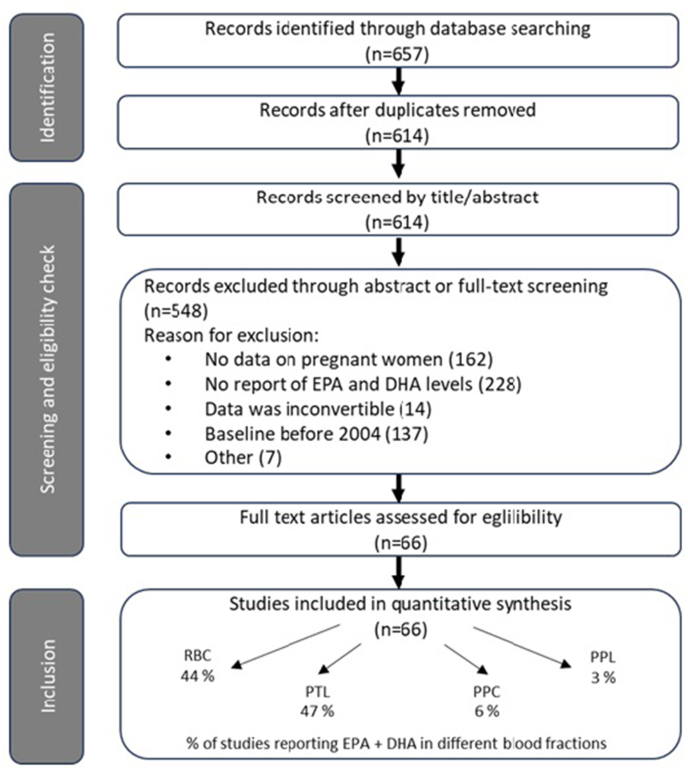
PRISMA flow diagram of the study selection process. Records identified through database searching (*n* = 657) were screened by title and abstract, and 591 were excluded. After full-text eligibility assessment, 66 studies were included in the analysis. The breakdown of included studies according to blood fraction was as follows: red blood cells (RBCs, 44%), plasma total lipids (PTLs, 47%), plasma phosphatidylcholine (PPC, 6%), and plasma phospholipids (PPLs, 3%).

The preliminary search yielded 657 references (Figure 1); 591 studies were excluded due to a failure to meet all pre-established inclusion and exclusion criteria. The 2 most common reasons for the exclusion of studies were the absence of reporting on EPA+DHA concentrations/the reporting of only EPA concentrations and not providing data on pregnant women. Although some studies had multiple reasons for exclusion, each study was assigned to a single exclusion category. Ultimately, 66 studies were included [[41], [42], [43], [44], [45], [46], [47], [48], [49], [50], [51], [52], [53], [54], [55], [56], [57], [58], [59], [60], [61], [62], [63], [64], [65], [66], [67], [68], [69], [70], [71], [72], [73], [74], [75], [76], [77], [78], [79], [80], [81], [82], [83], [84], [85], [86], [87], [88], [89], [90], [91], [92], [93], [94], [95], [96], [97], [98], [99], [100], [101], [102], [103], [104], [105], [106], [107]].

### Calculation of the eO3I

The extracted data were classified into categories corresponding to the blood fraction analyzed: PTL, PPL, PPC, and RBC. Serum data were considered equivalent to plasma, and RBC-PL data were consolidated under the RBC category. This categorization is supported by previous studies demonstrating comparable FA compositions between plasma and serum [108] and between RBC total lipid and RBC-PL [109].

Studies reported EPA+DHA concentrations as either relative percent [expressing EPA and DHA as weight% (wt%) or mol% of total FAs] or absolute concentration (expressing EPA and DHA by weight or moles per a given volume/weight); 72% of studies reported EPA+DHA concentrations as wt%, and 28% of studies reported levels as concentration.

To convert non–RBC-based EPA+DHA data to the eO3I levels [whenever % is used, it means % as a whole number, not a fraction (e.g. 4%, not 0.04)], we used the conversion equations developed by Schuchardt et al. [36] as follows:

• PTL EPA+DHA μmol/L to PTL EPA+DHA μg/mL: *y* = 0.317 × (*x*) + 0.8426.
• PTL EPA+DHA μg/mL to PTL EPA+DHA wt%: *y* = 0.0246 × (*x*) + 0.4451.
• PTL EPA+DHA wt% to RBC EPA+DHA wt%: *y* = 1.13 × (*x*) + 2.11.
• PPL EPA+DHA mol% to PPL EPA+DHA wt%: *y* = 1.1169 × (*x*) + 0.0016.
• PPL EPA+DHA wt% to RBC EPA+DHA wt%: *y* = 0.93 × (*x*) + 0.75.
• PPC EPA+DHA wt% to RBC EPA+DHA wt%: *y* = 0.6663 × (*x*) + 3.233.

The following conversion equations were newly developed by OmegaQuant Analytics, a commercial laboratory specializing in FA testing, based on their internal database:

• PTL DHA wt% to RBC EPA+DHA wt%: *y* = 2.1008 × (*x*) + 0.0142 (Supplementary Figure 1).
• RBC PL DHA wt% to RBC DHA wt%: *y* = 0.744 × (*x*) + 0.37 (Supplementary Figure 2).
• RBC DHA wt% to RBC EPA+DHA wt%: *y* = 1.3648 × (*x*) − 0.9379 (Supplementary Figure 3).

In certain instances, multiple equations were required to convert EPA and DHA, or DHA-only values, into eO3I concentrations. If median values and a measure of dispersion were reported, mean values were estimated [110].

### Calculation and categorization of the within-country eO3I levels and formation of the global map

A weighted mean of the eO3I was calculated for countries with several studies. For countries with only 1 study, the reported eO3I value was used for the map. To enable a direct comparison with the 2024 EPA+DHA world map [36], we applied the same cutoff ranges for the mean eO3I as used in previous studies [35,36] as follows:

• very low/undesirable (≤4%, red)
• low/insufficient (>4%–6%, orange)
• moderate/sufficient (>6%–8%, yellow)
• desirable (>8%, green).

The eO3I map was created using mapchart (https://www.mapchart.net/world.html).

### Statistical analysis and sensitivity analysis

To explore whether the conversion of non–RBC-based EPA+DHA and DHA-only metrics into eO3I might introduce systematic bias, we conducted a sensitivity comparison between studies reporting directly measured RBC values and those based on converted non–RBC data. The mean EPA+DHA concentrations of different studies were initially tested for normality using the Shapiro–Wilk test, visual histogram inspection, and evaluation of skewness and kurtosis. As the distribution of mean eO3I values was normal across cohorts, a Student *t* test was applied to compare the mean values from studies reporting directly measured RBC LC n–3 PUFA levels compared with those using converted non-RBC metrics.

## Results

### Characteristics of included studies

As many OSs as RCTs were included in the study. In total, data on 33,390 women from 28 countries were analyzed (Table 1). Most studies were conducted in Europe and North America. Almost half of all women studied come from Europe, and two-thirds of them came from the Netherlands.

**TABLE 1:** Demographics of included studies and studied subjects.

Characteristic	Studies, *n* (%)	Subjects, *n* (%)
Study type		
Observational studies	33 (50)	20,218 (61)
Randomized controlled trials	33 (50)	13,172 (39)
Region		
Asia	7 (11)	2471 (7)
Oceania	2 (3)	5843 (18)
Middle East	1 (1)	150 (<1)
Europe	28 (42)	15,837 (47)
North America	21 (32)	6844 (21)
Central and South America	3 (5)	285 (1)
Africa	4 (6)	1960 (6)

### Global distribution of the eO3I

**FIGURE 2:**
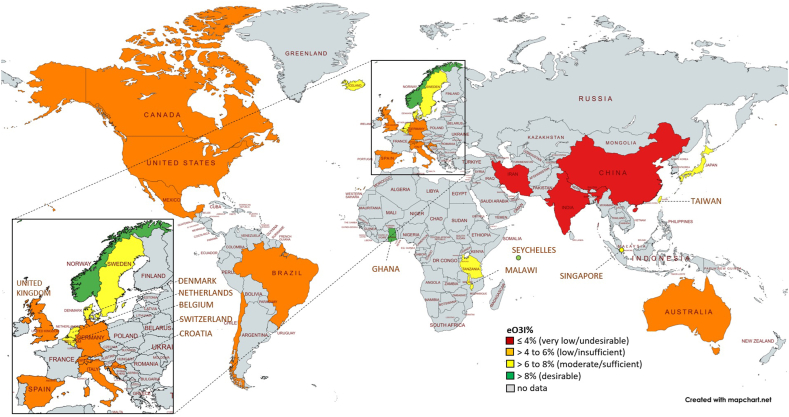
Global map of estimated Omega-3 Index (eO3I) in pregnant women. Global estimated red blood cell EPA+DHA concentrations obtained from observational studies and RCTs (baseline only) computed from different sample types (red blood cells or plasma lipids, i.e. total, phospholipid, or phosphatidylcholine) and measured in either concentrations or relative amounts. The mean eO3I of each country was categorized as very low/undesirable (≤4%, red), low/insufficient (>4%–6%, orange) moderate/sufficient (>6%–8%, yellow), or desirable (>8%, green). For countries where several studies were available, a weighted mean (by sample size) of eO3I was calculated.

The eO3I levels for the individual countries are shown in Table 2 and Figure 2. Detailed information on the eO3I levels from the individual studies, including the year of baseline/study data collection, study type, demographics, and blood fraction, is provided in Supplementary Table 2.

**TABLE 2:** Distribution of the estimated Omega-3 Index (eO3I) in different countries.

Region/country	*n*	eO3I (%), mean
**Asia**	2471	
China	555	2.46
India	354	2.38
Japan	566	6.69
Taiwan	36	6.50
Singapore	960	6.70
**Oceania**	5843	
Australia	5843	5.85
**Middle east**	150	
Iran^1^	150	2.50
**Europe**	15,837	
Belgium	71	6.06
Croatia	60	5.11
Denmark	699	7.65
Germany	415	5.81
Iceland	77	6.96
Italy	730	5.27
Netherlands	10,739	7.15
Norway	517	8.13
Spain	1428	5.95
Sweden	153	7.73
Switzerland	190	5.68
United Kingdom	758	5.01
**North America**	6844	
Canada	458	5.51
Mexico	198	4.43
USA	6188	5.46
**Central and South America**	285	
Brazil	245	5.27
Chile	40	5.50
**Africa**	1960	
Ghana	313	9.20
Malawi	315	7.70
Seychelles	1265	9.28
Tanzania	67	6.13

1 Geopolitically assigned to the Middle East, but located in Western Asia.

There are vast variations in the EPA+DHA status across the globe. Although 3 countries (China, Iran, and India) showed very low levels (≤4%, red), most countries fall into the low range (4%–6%, orange). This includes all North and South/Central American countries as well as Australia and most of central Europe. Moderate eO3I levels (>6%–8%, yellow) can be found in several Northern European countries (e.g. Sweden, Denmark, and Norway), and some east Asian (e.g. Japan, Singapore, and Taiwan) and African countries (e.g. Malawi and Tanzania). The highest eO3I levels in pregnant women were measured in the Seychelles (9.28%), Ghana (9.20%), and Norway (8.13%).

In countries with multiple studies, notable variability in mean eO3I values (≥2%) was observed, ranging from 3.45% to 5.36% in Brazil, 6.80% to 10.70% in Norway, 3.99% to 6.77% in the United Kingdom, and 3.39% to 7.26% in the United States.

### Agreement between direct RBC-based and converted non–RBC-based data

No statistically significant difference was observed between the mean eO3I values derived from direct RBC measurements and those estimated via conversion from non-RBC blood fractions (*P* > 0.05), indicating no evidence of systematic over- or underestimation due to the conversion approach at the group level (Table 3).

**TABLE 3:** Comparison of mean estimated Omega-3 Index (eO3I) values between studies with direct RBC-based data and those using converted blood concentrations of non–RBC-based data.

eO3I: RBC-based studies	eO3I: non–RBC-based studies	*t* test
5.59 ± 1.79	5.92 ± 1.64	*P* = 0.446

Values are mean ± SD.

Abbreviations: RBC, red blood cell.

## Discussion

Our analysis of EPA+DHA concentrations in pregnant women across the globe reveals a considerable lack of geographical diversity in the available data. Data from just >33,000 pregnant women in 28 countries are available, representing only 15% of all countries worldwide, highlighting significant global gaps in EPA+DHA status during pregnancy. Large regions including Central Asia, parts of Africa, South America, and the Middle East lack data on maternal EPA+DHA status. Furthermore, in countries where data are available, they are often derived from small cohorts, limiting generalizability. In 2 of the world’s most populous countries, China and India, data are available for only 555 and 354 individuals, respectively.

The eO3I categories are based on previous literature linking long-term EPA+DHA status with health outcomes [31,33,35,36]. The lower (<4%) and upper (>8%) cutoffs for RBC EPA+DHA were originally proposed for cardiovascular disease [33] and applied to other health outcomes [[111], [112], [113], [114], [115]]. Whether these categories also apply to pregnancy remains uncertain. Given the crucial role of DHA in fetal development, establishing maternal EPA+DHA (or DHA alone) thresholds would be useful in guiding dietary and supplemental advice. However, consensus pregnancy-specific targets are lacking. Proposals include >5% RBC DHA (∼5.9% EPA+DHA [116]), 8–11% RBC EPA+DHA [38], and <3.6% plasma ALA+EPA+DPAn–3+DHA as a marker for supplementation need [117]. Larger studies with standardized sampling windows are required to define pregnancy-specific thresholds. Excessively high intake may also present risks [[118], [119], [120]].

The overall EPA+DHA status appears inadequate in many regions. Almost all analyzed countries (except Ghana, Seychelles, and Norway) fall >8% eO3I; China, India, and Iran show an eO3I of <4%. Comparison with the 2024 EPA+DHA world map based on the general population [36] reveals similar geographic patterns, but the general population data set included 330,000 individuals from 48 countries, whereas our data set includes 33,390 pregnant women from 28 countries. Although the 2024 EPA+DHA world map included more Asian and European countries, the present pregnancy-specific analysis adds data from Singapore, Ghana, Malawi, Seychelles, Tanzania, and Chile. Both maps illustrate substantial evidence gaps for maternal EPA+DHA status.

In most countries, maternal EPA+DHA levels are similar to the general population [36]. Regional differences reflect dietary patterns. Very low EPA+DHA levels in China, India, and Iran align with historically low fish intake [121,122]. In traditionally high-fish-consuming countries like Japan and Iceland, lower eO3I in pregnant women may reflect lower fish intake and an ongoing Westernization of the diet among younger generations [[123], [124], [125], [126]]. Moreover, the difference may partly be attributed to age variations per se, as RBC EPA+DHA levels tend to increase with age [126]. Japan’s population map cohort was older (61 y) than the pregnancy cohort (∼32 y) [36,64]. In the Netherlands, pregnant women had higher eO3I (7.2%) than the general population (4.5%) [36], despite moderate fish intake [127], possibly reflecting greater supplement use.

Pregnant women in the Seychelles (9.28%) and Norway (8.13%) showed the highest eO3I, consistent with high fish intake [127]. Surprisingly, high levels in Ghana and Malawi [55,128] likely reflect local diets rich in small oily fish and proximity to water; however, these are small, localized samples and not necessarily nationally representative.

Although dietary EPA+DHA intake predicts status [36], national intake estimates cannot be directly applied to pregnant women. Pregnancy-specific restrictions [129], variation in supplement use [130], and changes in PUFA metabolism [131] also influence status. Differences between species, season, and preparation further complicate interpretation. Better dietary intake data in pregnancy are needed.

Despite unclear pregnancy-specific cutoffs, EPA+DHA concentrations appear suboptimal to critical for favorable pregnancy or maternal outcomes. From a public health perspective, it would be important to more effectively monitor the status of EPA+DHA in pregnant women worldwide using standardized blood sampling windows and uniform analytical methods. Many organizations advise that pregnant women consume an additional 200 to 300 mg/d of DHA above the general population recommendations to maintain adequate LC n–3 PUFA concentrations [[132], [133], [134]], yet actual intake remains low. For example, the mean intake of EPA and DHA among United States childbearing-age and pregnant women in the years 2001 to 2014 was 88 mg/d and supplementation is rare [135]. Higher intake may be required to maintain status due to maternal-fetal transfer [38,136].

Given the low maternal intake of EPA+DHA from fish and seafood in many countries, supplementation represents a feasible and potentially more effective strategy, as recently emphasized by an expert review panel in the *American Journal of Obstetrics and Gynecologists* [23]. Still, the blood concentration of EPA+DHA at which to provide high dose supplementation is yet to be decided upon. Based on the expert review panel proposal, EPA+DHA/DHA supplements should be made accessible and affordable for pregnant women (eg, through a doctor’s prescription or public funding), and supplementation guidelines should be incorporated into standard prenatal care protocols. Algal-based DHA supplements are particularly important for those following plant-based diets, as pregnancy-related concerns about mercury exposure often restrict fish consumption, making alternative EPA+DHA sources increasingly vital for this group [23].

### Strengths and limitations

Among the strengths of this study are the following: *1*) the inclusion of relatively recent blood samples (drawn in 2004 or later) providing contemporary insights into maternal EPA+DHA status; *2*) the incorporation of both observational and (baseline) RCT data, allowing for broader population coverage; and *3*) the inclusion of subjects regardless of health status or supplementation practices, providing a more realistic picture of maternal EPA+DHA status.

However, several limitations must be acknowledged. As noted earlier, sample sizes varied dramatically across different regions, ranging from as few as 36 participants in Taiwan to >10,739 in the Netherlands, and it is not clear that generalizing from the sample to the country is valid. Limited geographic representation within large countries, potential urban–rural sampling disparities (as observed in China), and socioeconomic factors were not consistently addressed across the various studies.

The blood collection periods for LC n–3 PUFA status analysis in the various studies were inconsistent (e.g. first, second, and third trimester). Given the dynamic nature of LC n–3 PUFA metabolism during pregnancy, the EPA+DHA blood status might vary by trimester/gestational weeks. However, based on the limited studies that assessed EPA+DHA status across all 3 trimesters [57,58,92], we observed no significant differences (data not shown).

It should be noted that the applied conversion equations were developed in nonpregnant individuals, and their validity under pregnancy-related metabolic conditions is currently unknown. Pregnancy is characterized not only by physiological hyperlipidemia but also by qualitative dyslipidemia [136], i.e. disproportionate alterations across lipid fractions that may, in principle, affect both absolute and relative EPA+DHA metrics. However, sensitivity analysis suggests that, at least at the group level, the conversion of non–RBC-based metrics does not produce an obvious systematic shift in mean eO3I levels. However, this approach cannot account for potential pregnancy-specific metabolic distortions at the individual level, and dedicated validation of conversion models in pregnant populations remains urgently needed.

Additionally, important confounding factors are not consistently reported such as dietary restrictions due to pregnancy-specific concerns, prepregnancy EPA+DHA status, and single compared with multiple fetuses. The EPA+DHA status can be influenced by various factors including education and socioeconomic status [137], urbanization [138], and proximity to (EPA+DHA-rich) fish sources [139]. Correspondingly, the variability in mean eO3I values observed between studies within the same country likely reflects differences in population characteristics and variation in dietary patterns and supplement use. This underscores the need for harmonized methodologies and more nationally representative sampling frameworks when interpreting country-level eO3I estimates. During pregnancy, these factors may be further complicated by variations in health care systems, prenatal care access, and national supplementation guidelines. As many studies do not report n–3 PUFA data stratified by these variables, ensuring representative estimates for each country’s pregnant population remains challenging.

## Conclusion

In conclusion, despite the many challenges to accurately assess the EPA+DHA status among pregnant women around the world, overall levels are clearly low. Thus, efforts should focus on improving EPA+DHA intake in pregnancy. Optimizing maternal EPA+DHA status requires a multifaceted approach that integrates advanced predictive models with targeted health policy interventions. The limited availability of data, with significant gaps particularly in Central Asia, large parts of Africa and South America, highlights the need for more comprehensive global surveillance. Notably, pregnant women in some countries show different eO3I patterns compared with general population data, suggesting that pregnancy-specific factors may modify the relationship between dietary intake and blood concentrations of EPA+DHA. These findings underscore the need for targeted interventions, including routine EPA+DHA status screening during pregnancy and the development of pregnancy-specific reference ranges. National health agencies should prioritize maternal EPA+DHA status assessment and improvement as part of their public health strategies, given its significant implications for both maternal and child health outcomes.

## Author contributions

The authors’ responsibilities were as follows — TD: performed investigation, formal analysis, data curation, and visualization and wrote the original draft; KHJ: reviewed and editing the manuscript; WSH: responsible for methodology, conceptualization, and review and editing of the manuscript; AH: reviewed and editing the manuscript and obtained resources; JPS: was responsible for methodology, conceptualization, investigation, visualization, validation, reviewed and editing the manuscript, funding acquisition, supervision, and project administration; and all authors: contributed to the final version and read and approved it for submission.

## Data availability

The datasets generated and analyzed for this study are available from the corresponding author on reasonable request. No restrictions apply beyond ensuring participant confidentiality.

## Declaration of generative AI and AI-assisted technologies in the writing process

During the preparation of this work, the authors used ChatGPT (OpenAI) and DeepL for language editing and improving the clarity of the text. After using these tools, the authors carefully reviewed and edited the content as needed and take full responsibility for the final content of the publication.

## Funding

The project was partly financed by the Global Organization for EPA and DHA Omega-3 (GOED). The sponsor had no role in the design of the study; the collection, analysis, or interpretation of the data; the writing of the manuscript; or the decision to submit the manuscript for publication.

## Conflicts of interest

WSH and KHJ hold stock in OmegaQuant Analytics, a laboratory that offers FA testing. The other authors declare no conflicts of interest.
