# Supplementary material for: Global Comparison of Erythrocyte EPA and DHA Concentrations in Pregnant Women
Source: J Nutr. 2025 Dec 27;156(2):101299. doi: 10.1016/j.tjnut.2025.101299 (PMC12975356; doi:10.1016/j.tjnut.2025.101299)
Supplement: Multimedia component 1 [file mmc1.pdf]

Supplementary Material

Title: Global Comparison of Erythrocyte EPA and DHA Concentrations in Pregnant Women

First Author: Tessa Deutsch

Correspondence:
Jan Philipp Schuchardt
Institute of Food and One Health, Faculty of Natural Sciences, Leibniz University Hannover
Email: schuchardt@foh.uni-hannover.de

Supplementary Figure 1: Association between DHA wt% in plasma total lipids (PTL) and EPA+DHA wt% in red blood cells (RBC); (n=100, R^2=0.7699). The formula for converting PTL DHA wt% to RBC EPA+DHA wt% derived from the graph is: y=2.1008*(x)+0.0142. Abbreviations: DHA – Docosahexaenoic acid; EPA – Eicosapentaenoic acid.

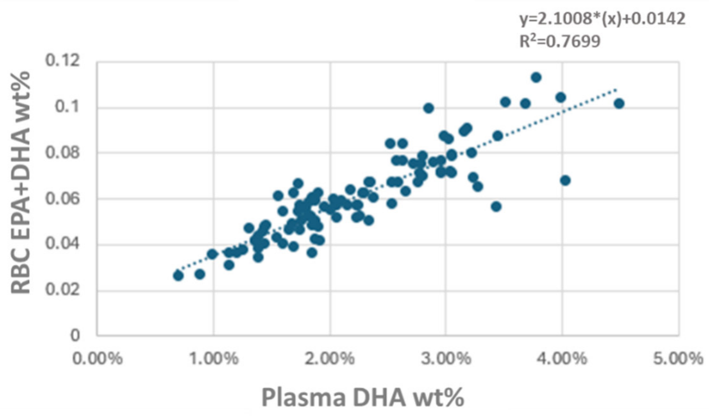

Supplementary Figure 2: Association between DHA wt% in red blood cell (RBC) phospholipids (PL) and DHA wt% in RBC; (n=20, R^2=0.9367). The formula for converting RBC PL DHA wt% to RBC DHA wt% derived from the graph is: y=0.744*(x)+0.37. Abbreviations: DHA – Docosahexaenoic acid; EPA – Eicosapentaenoic acid.

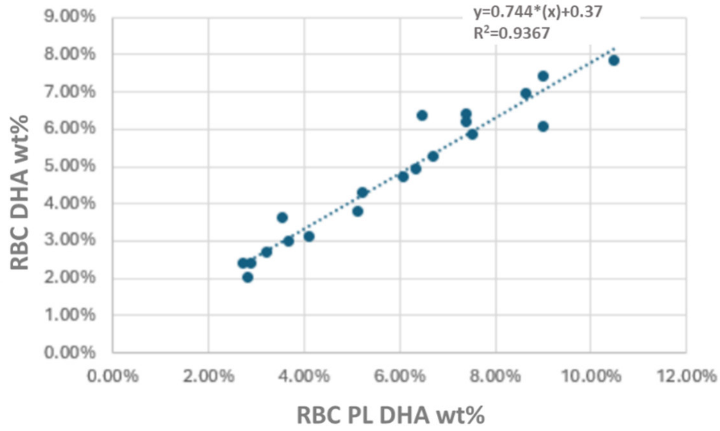

Supplementary Figure 3: Association between DHA wt% in in red blood cells (RBC) and EPA+DHA wt% in RBC; (n=100, R^2=0.9318). The formula for converting RBC DHA wt% to RBC EPA+DHA wt% derived from the graph is: y=1.3648*x-0.9379. Abbreviations: DHA – Docosahexaenoic acid; EPA – Eicosapentaenoic acid.

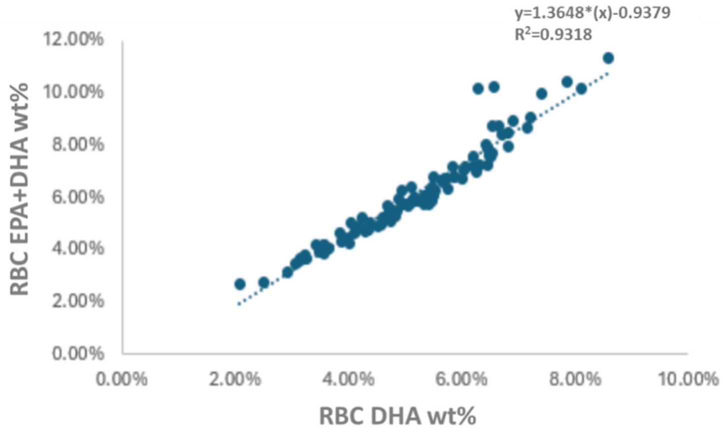

Supplementary Table 1: Search Strategy.

Search	Database	Terms	Records
1	GOED	("pregnant women" OR "pregnancy" OR "prenatal" OR "maternal") OR ("lactating women" OR "lactation" OR "breastfeeding" OR "postpartum") AND ("omega-3" OR "n 3 fatty acids" OR "EPA" OR "DHA" OR "docosahexa*" OR "eicosapent*" OR "fatty acids" OR "omega 3 fatty acids") AND ("index" OR "levels" OR "concentration" OR "status" OR "measurement" OR "assessment") AND ("clinical trial"[Publication Type] OR "randomized controlled trial"[Publication Type] OR "observational study"[Publication Type]) AND ("2003/01/01"[Date - Publication] : "2025/05/31"[Date - Publication]) AND ("english"[Language]) NOT ("review"[Publication Type] OR "meta-analysis"[Publication Type])	149
2	PubMed	("pregnant women" OR "pregnancy" OR "prenatal" OR "maternal") OR ("lactating women" OR "lactation" OR "breastfeeding" OR "postpartum") AND ("omega-3" OR "n 3 fatty acids" OR "EPA" OR "DHA" OR "docosahexa*" OR "eicosapent*" OR "fatty acids" OR "omega 3 fatty acids") AND ("index" OR "levels" OR "concentration" OR "status" OR "measurement" OR "assessment") AND ("clinical trial"[Publication Type] OR "randomized controlled trial"[Publication Type] OR "observational study"[Publication Type]) AND ("2003/01/01"[Date - Publication] : "2025/05/31"[Date - Publication]) AND ("english"[Language]) NOT ("review"[Publication Type] OR "meta-analysis"[Publication Type])	508

Abbreviations: DHA – Docosahexaenoic acid; EPA – Eicosapentaenoic acid.

Supplementary Table 2: Global distribution of the mean estimated Omega-3 Index (eO3I) in different studies worldwide (some studies include EPA+DHA data from different countries/regions. Hence, several references appear multiple times).

Country	Year of baseline/ blood draw	Study type	Participants (n)	Age (study) mean	Trimester	Blood fraction	eO3I (%)
**Australia**			5843 (total)				**5.85 (mean)**
(Parker et al., 2015)	N/A	RCT	773	31.0	3	RBC	6.68
(Simmonds et al., 2020)	2013-2017	RCT	5070	N/A	1;2	PTL	5.73
**Belgium**							
(Hoge et al., 2019)	2016	OS	71	29.0	1;2	RBC	**6.06**
**Brazil**			245 (total)				**5.27 (mean)**
(Vaz et al., 2014)	2009-2011	OS	234	N/A	1	PTL	5.36
(Ribeiro et al., 2011)	2009-2011	RCT	11	N/A	3	PTL	3.45
**Canada**			458 (total)				**5.51 (mean)**
(Zhao et al., 2015)	2006-2008	OS	140	31.5	2;3	PTL	6.85
(Friesen and Innis, 2010)	N/A	OS	105	N/A	3	PPC	4.85
(Mulder et al., 2014)	2004-2008	RCT	213	33.0	2	PPC	4.96
**Chile**							
(Valenzuela et al., 2015)	2012-2013	RCT	40	28.6	2	RBC	**5.50**
**China**							
(He et al., 2020)	2014-2017	OS	555	27.4	3	PTL	**2.46**
**Croatia**							
(Djelmis et al., 2018)	2012-2016	OS	60	30.9	3	PTL	**5.11**
**Denmark**							
(Vinding et al., 2019)	2008-2010	RCT	699	32.2	2	PTL	**7.65**
**Germany**			415 (total)				**5.81 (mean)**
(Gellert et al., 2016)	2013-2015	OS	213	30.4	3	RBC	6.62
(Hauner et al., 2012)	2006-2009	RCT	202	31.7	2	RBC	4.96
**Ghana**							
(Oaks et al., 2017)	2011-2012	RCT	313	26.5	1;2	PTL	**9.20**
**Iceland**							
(Magnusardottir et al., 2009)	N/A	OS	77	27.4	1	RBC	**6.96**
**India**			354 (total)				**2.38 (mean)**
(Meher et al., 2016)	N/A	OS	99	23.3	1;2;3	RBC	3.16
(Muthayya et al., 2009)	2002-2006	OS	122	24.0 (median)	1;2;3	RBC	2.11
(Dwarkanath et al., 2009)	2002-2006	OS	133	24.7	1	RBC	2.05

**Iran**							
(Farshbaf-Khalil et al., 2017)	2014	RCT	150	26.5	2	PTL	**2.50**
**Italy**			730 (total)				**5.27 (mean)**
(Massari et al., 2020)	2016-2019	RCT	141	31.9	2	RBC	6.61
(Valent et al., 2013)	2007-2009	OS	589	33.3		PTL	4.95
**Japan**							
(Hamazaki et al., 2016)	2011-2014	OS	566	31.5	2	PTL	**6.69**
**Malawi**							
(Oaks et al., 2017)	2011-2012	RCT	315	24.9	1;2	PTL	**7.70**
**Mexico**							
(Imhoff-Kunsch et al., 2011)	2005-2007	RCT	198	26.1	2	PTL	**4.43**
**Netherlands**			10739 (total)				**7.15 (mean)**
(Stoutjesdijk et al., 2018)	N/A	RCT	36	31.0	2	RBC	5.61
(Voortman et al., 2018)	2002-2006	OS	6999	30.4	2	PTL	7.88
(van Eijsden et al., 2008)	2003-2004	OS	3704	N/A	1;2	PPL	5.79
**Norway**			517 (total)				**8.13 (mean)**
(Markhus et al., 2015)	2009-2011	OS	69	30.0	3	RBC	6.80
(Araujo et al., 2020)	2011-2012	OS	247	30.1	2	RBC	7.70
(Brantsaeter et al., 2010)	2003-2004	OS	119	31.2	2	RBC	10.70
(Braarud et al., 2018)	2009-2011	OS	82	31.0	3	RBC	6.80
**Seychelles**							
(Strain et al., 2015)	2008-2011	OS	1265	26.9	3	PTL	**9.28**
**Singapore**							
(Yu et al., 2018)	2009-2010	OS	960	30.3	3	PPC	**6.70**
**Spain**			1428 (total)				**5.95 (mean)**
(Aparicio et al., 2021)	2013-?	OS	479	30.6	1;3	PTL	5.00
(Montes et al., 2013)	2004-2006	OS	211	31.7	1	PTL	5.54
(Gázquez et al., 2021)	2015-2018	OS	560	33.2	2	PTL	6.68
(Hurtado et al., 2015)	2009-2010	RCT	110	30.2	3	PTL	6.72
(Prieto-Sánchez et al., 2017)	N/A	OS	68	32.9	3	PTL	6.64
**Sweden**			153 (total)				**7.63 (mean)**
(Bosaeus et al., 2015)	2009-2012	RCT	35	31.3	1	PTL	7.33
(Warstedt et al., 2009)	2003-2005	RCT	118	31.6	3	PPL	7.72
**Switzerland**			190 (total)				**5.68 (mean)**
(Bandres-Meriz et al., 2021)	2017-2018	OS	123	31.4	1	PTL	5.80
(Urech et al., 2020)	N/A	OS	67	31.8	3	RBC	5.45
**Taiwan**							
(Su et al., 2008)	2004-2006	RCT	36	31.0	2	RBC	**6.50**

**Tanzania**							
(Kuipers et al., 2011)	N/A	OS	67	24.3	3	RBC	**6.13**
**UK**			758 (total)				**5.01 (mean)**
(Goodfellow et al., 2021)	2012-2017	OS	264	30.4	2	PTL	3.99
(Min et al., 2016)	2007-2012	RCT	138	31.5	2;3	PPC	6.77
(Garcia-Rodriguez et al., 2017)	2007-2012	RCT	65	N/A	2	RBC	4.44
(Ogundipe et al., 2016)	N/A	RCT	291	33.2	1	RBC	5.23
**USA**			6188 (total)				**5.46 (mean)**
(Klebanoff et al., 2011)	2005-2006	OS	701	27.5	2	RBC	3.39
(Haghiac et al., 2015)	2009-2011	RCT	49	27.0	1;2	PTL	5.73
(Powell et al., 2021)	N/A	OS	32	29.9	2	PTL	3.84
(Monthé-Drèze et al., 2018)	2005-2006	RCT	532	27.0	2	PTL	6.52
(Rosa et al., 2020)	2006-2011	OS	1019	N/A	2	PTL	6.90
(Mozurkewich et al., 2013)	2008-2011	RCT	125	30.3	2	PTL	7.26
(Huang et al., 2020)	2006-2019	OS	218	34.1	3	PTL	4.94
(Colombo et al., 2019)	N/A	RCT	263	N/A	2	RBC	3.92
(Lager et al., 2017)	N/A	RCT	38	28.2	3	RBC	7.11
(Gustafson et al., 2013)	2009-2011	RCT	67	25.5	2	RBC	4.04
(Carlson et al., 2013)	2006-2009	RCT	350	25.1	1;2	RBC	3.93
(Judge et al., 2012)	N/A	RCT	40	23.9	2	RBC	6.11
(Foster et al., 2017)	N/A	RCT	63	29.3	3	RBC	7.11
(Oken et al., 2013)	2010	RCT	55	30.9	2	PTL	4.08
(Valentine et al., 2021)	2016-2020	RCT	902	N/A	2	RBC	6.11
(Harris et al., 2015)	N/A	RCT	634	24.3	2	RBC	3.91
(Carlson et al., 2021)	2016-2020	RCT	1100	30	2	RBC	6.05

Abbreviations: DHA – Docosahexaenoic acid; EPA – Eicosapentaenoic acid; N/A – Not Available; OS – Observational Study; PPC – Plasma Phosphatidylcholine; PTL – Plasma Total Lipids; RBC – Red Blood Cells; RCT – Randomized Controlled Trial.

REFERENCES

Aparicio, E., Martín-Grau, C., Bedmar, C., Serrat Orus, N. S., Basora, J., Arija, V., et al. (2021). Maternal Factors Associated with Levels of Fatty Acids, Specifically n-3 PUFA during Pregnancy: ECLIPSES Study. *Nutrients* 13, 317.

Araujo, P., Kjellevold, M., Nerhus, I., Dahl, L., Aakre, I., Moe, V., et al. (2020). Fatty Acid Reference Intervals in Red Blood Cells among Pregnant Women in Norway-Cross Sectional Data from the 'Little in Norway' Cohort. *Nutrients* 12. doi: 10.3390/nu12102950

Bandres-Meriz, J., Majali-Martinez, A., Hoch, D., Morante, M., Glasner, A., van Poppel, M., et al. (2021). Maternal C-Peptide and Insulin Sensitivity, but Not BMI, Associate with Fatty Acids in the First Trimester of Pregnancy. *International Journal of Molecular Sciences* 22, 10422.

Bosaeus, M., Hussain, A., Karlsson, T., Andersson, L., Hulthén, L., Svelander, C., et al. (2015). A randomized longitudinal dietary intervention study during pregnancy: effects on fish intake, phospholipids, and body composition. *Nutrition Journal* 14, 1.

Braarud, H. C., Markhus, M. W., Skotheim, S., Stormark, K. M., Frøyland, L., Graff, I. E., et al. (2018). Maternal DHA Status during Pregnancy Has a Positive Impact on Infant Problem Solving: A Norwegian Prospective Observation Study. *Nutrients* 10. doi: 10.3390/nu10050529

Brantsaeter, A. L., Haugen, M., Thomassen, Y., Ellingsen, D. G., Ydersbond, T. A., Hagve, T.-A., et al. (2010). Exploration of biomarkers for total fish intake in pregnant Norwegian women. *Public Health Nutr* 13, 54–62. doi: 10.1017/S1368980009005904

Carlson, S. E., Colombo, J., Gajewski, B. J., Gustafson, K. M., Mundy, D., Yeast, J., et al. (2013). DHA supplementation and pregnancy outcomes. *American Journal of Clinical Nutrition* 97, 808–815.

Carlson, S. E., Gajewski, B. J., Valentine, C. J., Kerling, E. H., Weiner, C. P., Cackovic, M., et al. (2021). Higher dose docosahexaenoic acid supplementation during pregnancy and early preterm birth: A randomised, double-blind, adaptive-design superiority trial. *EClinicalMedicine* 36, 100905. doi: 10.1016/j.eclinm.2021.100905

Colombo, J., Shaddy, D. J., Gustafson, K., Gajewski, B. J., Thodosoff, J. M., Kerling, E., et al. (2019). The Kansas University DHA Outcomes Study (KUDOS) clinical trial: long-term behavioral follow-up of the effects of prenatal DHA supplementation. *The American Journal of Clinical Nutrition* 109, 1380–1392.

Djelmis, J., Ivaniševic, M., Desoye, G., van Poppel, M., Berberovic, E., Soldo, D., et al. (2018). Higher Cord Blood Levels of Fatty Acids in Pregnant Women With Type 1 Diabetes Mellitus. *The Journal of Clinical Endocrinology & Metabolism* 103, 2620–2629.

Dwarkanath, P., Muthayya, S., Thomas, T., Vaz, M., Parikh, P., Mehra, R., et al. (2009). Polyunsaturated fatty acid consumption and concentration among South Indian women during pregnancy. *Asia Pac J Clin Nutr* 18, 389–394.

Farshbaf-Khalil, A., Mohamad-Alizadeh, S., Darabi, M., Hematzadeh, S., Mehdizadeh, A., Shaaker, M., et al. (2017). The effect of fish oil supplementation on serum phospholipid fatty acids profile during pregnancy: A double blind randomized controlled trial. *Women Health* 57, 137–153.

Foster, B. A., Escaname, E., Powell, T. L., Larsen, B., Siddiqui, S. K., Menchaca, J., et al. (2017). Randomized Controlled Trial of DHA Supplementation during Pregnancy: Child Adiposity Outcomes. *Nutrients* 9, 566.

Friesen, R. W., and Innis, S. M. (2010). Linoleic acid is associated with lower long-chain n–6 and n–3 fatty acids in red blood cell lipids of Canadian pregnant women. *The American Journal of Clinical Nutrition* 91, 23–31.

Garcia-Rodriguez, C. E., Olza, J., Mesa, M. D., Aguilera, C. M., Miles, E. A., Noakes, P. S., et al. (2017). Fatty acid status and antioxidant defense system in mothers and their newborns after salmon intake during late pregnancy. *Nutrition* 33, 157–162.

Gázquez, A., Giménez-Bañón, M. J., Prieto-Sánchez, M. T., Martínez-Graciá, C., Suárez, C., Santaella-Pascual, M., et al. (2021). Self-Reported DHA Supplementation during Pregnancy and Its Association with Obesity or Gestational Diabetes in Relation to DHA Concentration in Cord and Maternal Plasma: Results from NELA, a Prospective Mother-Offspring Cohort. *Nutrients* 13, 843.

Gellert, S., Schuchardt, J. P., and Hahn, A. (2016). Higher omega-3 index and DHA status in pregnant women compared to lactating women – Results from a German nation-wide cross-sectional study. *Prostaglandins, Leukotrienes and Essential Fatty Acids* 109, 22–28.

Goodfellow, L., Care, A., Harrold, J., Sharp, A., Ivandic, J., Poljak, B., et al. (2021). Plasma long-chain omega-3 fatty acid status and risk of recurrent early spontaneous preterm birth: a prospective observational study. *Acta Obstetricia et Gynecologica Scandinavica* 100, 1401–1411.

Gustafson, K. M., Carlson, S. E., Colombo, J., Yeh, H.-W., Shaddy, D. J., Li, S., et al. (2013). Effects of docosahexaenoic acid supplementation during pregnancy on fetal heart rate and variability: a randomized clinical trial. *Prostaglandins, Leukotrienes and Essential Fatty Acids* 88, 331–338.

Haghiac, M., Yang, X., Presley, L., Smith, S., Dettelback, S., Minium, J., et al. (2015). Dietary Omega-3 Fatty Acid Supplementation Reduces Inflammation in Obese Pregnant Women: A Randomized Double-Blind Controlled Clinical Trial. *PLoS One* 10, e0137309.

Hamazaki, K., Harauma, A., Otaka, Y., Moriguchi, T., and Inadera, H. (2016). Serum n-3 polyunsaturated fatty acids and psychological distress in early pregnancy: Adjunct Study of Japan Environment and Children’s Study. *Translational Psychiatry* 6, e737.

Harris, M. A., Reece, M. S., McGregor, J. A., Wilson, J. W., Burke, S. M., Wheeler, M., et al. (2015). The Effect of Omega-3 Docosahexaenoic Acid Supplementation on Gestational Length: Randomized Trial of Supplementation Compared to Nutrition Education for Increasing n-3 Intake from Foods. *Biochemistry Research International*, 123078.

Hauner, H., Much, D., Vollhardt, C., Brunner, S., Schmid, D., Sedlmeier, E., et al. (2012). Effect of reducing the n-6:n-3 long-chain PUFA ratio during pregnancy and lactation on infant adipose tissue growth within the first year of life: an open-label randomized controlled trial. *The American Journal of Clinical Nutrition* 95, 383–394.

He, X., Dai, R., Tian, C., and Hu, C. (2020). Neurodevelopmental outcome at 1 year in offspring of women with gestational diabetes mellitus. *Gynecological Endocrinology*, 88–92.

Hoge, A., Tabar, V., Donneau, A. F., Dardenne, N., Degée, S., Timmermans, M., et al. (2019). Imbalance between Omega-6 and Omega-3 Polyunsaturated Fatty Acids in Early Pregnancy Is Predictive of Postpartum Depression in a Belgian Cohort. *Nutrients* 11, 876.

Huang, Y., Iosif, A., Hansen, R. L., and Schmidt, R. J. (2020). Maternal polyunsaturated fatty acids and risk for autism spectrum disorder in the MARBLES high-risk study. *Autism* 24, 1191–1200.

Hurtado, J. A., Iznaola, C., Peña, M., Ruíz, J., Peña-Quintana, L., Kajarabille, N., et al. (2015). Effects of Maternal Ω-3 Supplementation on Fatty Acids and on Visual and Cognitive Development. *Journal of Pediatric Gastroenterology and Nutrition* 61, 472–480.

Imhoff-Kunsch, B., Stein, A. D., Villalpando, S., Martorell, R., and Ramakrishnan, U. (2011). Docosahexaenoic acid supplementation from mid-pregnancy to parturition influenced breast milk fatty acid concentrations at 1 month postpartum in Mexican women. *The Journal of Nutrition* 141, 321–326.

Judge, M. P., Cong, X., Harel, O., Courville, A. B., and Lammi-Keefe, C. J. (2012). Maternal consumption of a DHA-containing functional food benefits infant sleep patterning: an early neurodevelopmental measure. *Early Human Development* 88, 531–537.

Klebanoff, M. A., Harper, M., Lai, Y., Thorp, J. JR., Sorokin, Y., Varner, M. W., et al. (2011). Fish consumption, erythrocyte fatty acids, and preterm birth. *Obstetrics & Gynecology* 117, 1071–1077.

Kuipers, R. S., Luxwolda, M. F., Sango, W. S., Kwesigabo, G., Dijck-Brouwer, D. A., and Muskiet, F. A. (2011). Maternal DHA Equilibrium during Pregnancy and Lactation Is Reached at an Erythrocyte DHA Content of 8 g/100 g Fatty Acids. *The Journal of Nutrition* 141, 418–427.

Lager, S., Ramirez, V. I., Acosta, O., Meireles, C., Miller, E., Gaccioli, F., et al. (2017). Docosahexaenoic Acid Supplementation in Pregnancy Modulates Placental Cellular Signaling and Nutrient Transport Capacity in Obese Women. *The Journal of Clinical Endocrinology & Metabolism* 102, 4557–4567.

Magnusardottir, A. R., Steingrimsdottir, L., Thorgeirsdottir, H., Hauksson, A., and Skuladottir, G. V. (2009). Red blood cell n-3 polyunsaturated fatty acids in first trimester of pregnancy are inversely associated with placental weight. *Acta Obstetricia et Gynecologica Scandinavica* 88, 91–97.

Markhus, M. W., Rasinger, J. D., Malde, M. K., Frøyland, L., Skotheim, S., Braarud, H. C., et al. (2015). Docosahexaenoic Acid Status in Pregnancy Determines the Maternal Docosahexaenoic Acid Status 3-, 6- and 12 Months Postpartum. Results from a Longitudinal Observational Study. *PLoS One* 10, e0136409.

Massari, M., Novielli, C., Mandò, C., Di Francesco, S., Della Porta, M., Cazzola, R., et al. (2020). Multiple Micronutrients and Docosahexaenoic Acid Supplementation during Pregnancy: A Randomized Controlled Study. *Nutrients* 12, 2432.

Meher, A., Randhir, K., Mehendale, S., Wagh, G., and Joshi, S. (2016). Maternal Fatty Acids and Their Association with Birth Outcome: A Prospective Study. *PLoS One* 11, e0147359.

Min, Y., Djahanbakhch, O., Hutchinson, J., Eram, S., Bhullar, A. S., Namugere, I., et al. (2016). Efficacy of docosahexaenoic acid-enriched formula to enhance maternal and fetal blood docosahexaenoic acid levels: Randomized double-blinded placebo-controlled trial of pregnant women with gestational diabetes mellitus. *Clinical Nutrition* 35, 608–614.

Montes, R., Chisaguano, A. M., Castellote, A. I., Morales, E., Sunyer, J., and López-Sabater, M. C. (2013). Fatty-acid composition of maternal and umbilical cord plasma and early childhood atopic eczema in a Spanish cohort. *European Journal of Clinical Nutrition* 67, 658–663.

Monthé-Drèze, C., Penfield-Cyr, A., Smid, M. C., and Sen, S. (2018). Maternal Pre-Pregnancy Obesity Attenuates Response to Omega-3 Fatty Acids Supplementation During Pregnancy. *Nutrients* 10, 1908.

Mozurkewich, E. L., Clinton, C. M., Chilimigras, J. L., Hamilton, S. E., Allbaugh, L. J., Berman, D. R., et al. (2013). The Mothers, Omega-3, and Mental Health Study: a double-blind, randomized controlled trial. *American Journal of Obstetrics & Gynecology* 208, 313.e1-9.

Mulder, K. A., King, D. J., and Innis, S. M. (2014). Omega-3 fatty acid deficiency in infants before birth identified using a randomized trial of maternal DHA supplementation in pregnancy. *PLoS One* 9, e83764.

Muthayya, S., Dwarkanath, P., Thomas, T., Ramprakash, S., Mehra, R., Mhaskar, A., et al. (2009). The effect of fish and omega-3 LCPUFA intake on low birth weight in Indian pregnant women. *European Journal of Clinical Nutrition* 63, 340–346.

Oaks, B. M., Young, R. R., Adu-Afarwuah, S., Ashorn, U., Jackson, K. H., Lartey, A., et al. (2017). Effects of a lipid-based nutrient supplement during pregnancy and lactation on maternal plasma fatty acid status and lipid profile: Results of two randomized controlled trials. *Prostaglandins, Leukotrienes and Essential Fatty Acids* 117, 28–35.

Ogundipe, E., Johnson, M. R., Wang, Y., and Crawford, M. A. (2016). Peri-conception maternal lipid profiles predict pregnancy outcomes. *Prostaglandins, Leukotrienes and Essential Fatty Acids* 114, 35–43.

Oken, E., Guthrie, L. B., Bloomingdale, A., Platek, D. N., Price, S., Haines, J., et al. (2013). A pilot randomized controlled trial to promote healthful fish consumption during pregnancy: The Food for Thought Study. *Nutrition Journal* 12, 33.

Parker, G., Hegarty, B., Granville-Smith, I., Ho, J., Paterson, A., Gokiert, A., et al. (2015). Is essential fatty acid status in late pregnancy predictive of post-natal depression? *Acta Psychiatrica Scandinavica* 131, 148–156.

Powell, T. L., Barner, K., Madi, L., Armstrong, M., Manke, J., Uhlson, C., et al. (2021). Sex-specific responses in placental fatty acid oxidation, esterification and transfer capacity to maternal obesity. *Biochimica et Biophysica Acta (BBA) - Molecular and Cell Biology of Lipids* 1866, 158861.

Prieto-Sánchez, M. T., Ruiz-Palacios, M., Blanco-Carnero, J. E., Pagan, A., Hellmuth, C., Uhl, O., et al. (2017). Placental MFSD2a transporter is related to decreased DHA in cord blood of women with treated gestational diabetes. *Clinical Nutrition* 36, 513–521.

Ribeiro, P. D., Carvalho, F. D. F., Abreu, A. A., Sant'anna, M. T., Lima, R. J., and Carvalho, P. O. (2011). Effect of fish oil supplementation in pregnancy on the fatty acid composition of erythrocyte phospholipids and breast milk lipids. *International Journal of Food Sciences and Nutrition* 63, 36–40.

Rosa, M. J., Hartman, T. J., Adgent, M., Gardner, K., Gebretsadik, T., Moore, P. E., et al. (2020). Prenatal polyunsaturated fatty acids and child asthma: Effect modification by maternal asthma and child sex. *Journal of Allergy and Clinical Immunology* 145, 800 - 807.e4.

Simmonds, L. A., Sullivan, T. R., Skubisz, M., Middleton, P. F., Best, K. P., Yelland, L. N., et al. (2020). Omega-3 fatty acid supplementation in pregnancy-baseline omega-3 status and early preterm birth: exploratory analysis of a randomised controlled trial. *British journal of obstetrics and gynaecology* 127, 975–981.

Stoutjesdijk, E., Schaafsma, A., Dijck-Brouwer, D., and Muskiet, F. (2018). Fish oil supplemental dose needed to reach 1g% DHA+EPA in mature milk. *Prostaglandins, Leukotrienes and Essential Fatty Acid* 128, 53–61.

Strain, J. J., Yeates, A. J., van Wijngaarden, E., Thurston, S. W., Mulhern, M. S., McSorley, E. M., et al. (2015). Prenatal exposure to methyl mercury from fish consumption and polyunsaturated fatty acids: associations with child development at 20 mo of age in an observational study in the Republic of Seychelles. *The American Journal of Clinical Nutrition* 101, 530–537.

Su, K. P., Huang, S. Y., Chiu, T. H., Huang, K. C., Huang, C. L., Chang, H. C., et al. (2008). Omega-3 fatty acids for major depressive disorder during pregnancy: Results from a randomized, double-blind, placebo-controlled trial. *The Journal of Clinical Psychiatry* 69, 644–651.

Urech, C., Eussen, S., Alder, J., Stahl, B., Boehm, G., Bitzer, J., et al. (2020). Levels of n-3 and n-6 Fatty Acids in Maternal Erythrocytes during Pregnancy and in Human Milk and Its Association with Perinatal Mental Health. *Nutrients* 12, 2773.

Valent, F., Mariuz, M., Bin, M., Little, D., Mazej, D., Tognin, V., et al. (2013). Associations of prenatal mercury exposure from maternal fish consumption and polyunsaturated fatty acids with child neurodevelopment: a prospective cohort study in Italy. *Journal of Epidemiology* 23, 360–370.

Valentine, C. J., Khan, A. Q., Brown, A. R., Sands, S. A., DeFranco, E. A., Gajewski, B. J., et al. (2021). Higher-Dose DHA Supplementation Modulates Immune Responses in Pregnancy and Is Associated with Decreased Preterm Birth. *Nutrients* 13, 4248.

Valenzuela, R., Bascuñán, K. A., Chamorro, R., Barrera, C., Sandoval, J., Puigrredon, C., et al. (2015). Modification of Docosahexaenoic Acid Composition of Milk from Nursing Women Who Received Alpha Linolenic Acid from Chia Oil during Gestation and Nursing. *Nutrients* 7, 6405–6424.

van Eijsden, M., Hornstra, G., van der Wal, M. F., Vrijkotte, T. G., and Bonsel, G. J. (2008). Maternal n-3, n-6, and trans fatty acid profile early in pregnancy and term birth weight: a prospective cohort study. *The American Journal of Clinical Nutrition* 87, 887–895.

Vaz, J. S., Kac, G., Nardi, A. E., and Hibbeln, J. R. (2014). Omega-6 fatty acids and greater likelihood of suicide risk and major depression in early pregnancy. *Journal of Affective Disorders* 152-154, 76–82.

Vinding, R. K., Stokholm, J., Sevelsted, A., Chawes, B. L., Bønnelykke, K., Barman, M., et al. (2019). Fish Oil Supplementation in Pregnancy Increases Gestational Age, Size for Gestational Age, and Birth Weight in Infants: A Randomized Controlled Trial. *The Journal of Nutrition* 149, 628–634.

Voortman, T., Tielemans, M. J., Stroobant, W., Schoufour, J. D., Kiefte-de Jong, J. C., Steenweg-de Graaff, J., et al. (2018). Plasma fatty acid patterns during pregnancy and child's growth, body composition, and cardiometabolic health: The Generation R Study. *Clinical Nutrition* 37, 984–992.

Warstedt, K., Furuhjelm, C., Duchén, K., Fälth-Magnusson, K., and Fagerås, M. (2009). The effects of omega-3 fatty acid supplementation in pregnancy on maternal eicosanoid, cytokine, and chemokine secretion. *Pediatric Research* 66, 212–217.

Yu, Y. M., Chan, Y. H., Calder, P. C., Hardjojo, A., Soh, S. E., Lim, A. L., et al. (2018). Maternal PUFA status and offspring allergic diseases up to the age of 18 months. *British Journal of Nutrition* 113, 975–983.

Zhao, J. P., Levy, E., Shatenstein, B., Fraser, W. D., Julien, P., Montoudis, A., et al. (2015). Longitudinal circulating concentrations of long-chain polyunsaturated fatty acids in the third trimester of pregnancy in gestational diabetes. *Diabetic Medicine* 33, 939–946.
